# Sensorimotor Learning: Neurocognitive Mechanisms and Individual Differences

**DOI:** 10.1186/s12984-017-0279-1

**Published:** 2017-07-13

**Authors:** R. D. Seidler, R. G. Carson

**Affiliations:** 1 University of Florida, P.O. Box 118205, Gainesville, FL 32611-8205 USA; 2 Trinity College Dublin, Dublin, Ireland; 3 Queen’s University Belfast, Belfast, Ireland

**Keywords:** Motor learning, Adaptation, BANCOM, SNP, Genetic predictors, Genotype

## Abstract

Here we provide an overview of findings and viewpoints on the mechanisms of sensorimotor learning presented at the 2016 Biomechanics and Neural Control of Movement (BANCOM) conference in Deer Creek, OH. This field has shown substantial growth in the past couple of decades. For example it is now well accepted that neural systems outside of primary motor pathways play a role in learning. Frontoparietal and anterior cingulate networks contribute to sensorimotor adaptation, reflecting strategic aspects of exploration and learning. Longer term training results in functional and morphological changes in primary motor and somatosensory cortices. Interestingly, re-engagement of strategic processes once a skill has become well learned may disrupt performance.

Efforts to predict individual differences in learning rate have enhanced our understanding of the neural, behavioral, and genetic factors underlying skilled human performance. Access to genomic analyses has dramatically increased over the past several years. This has enhanced our understanding of cellular processes underlying the expression of human behavior, including involvement of various neurotransmitters, receptors, and enzymes. Surprisingly our field has been slow to adopt such approaches in studying neural control, although this work does require much larger sample sizes than are typically used to investigate skill learning. We advocate that individual differences approaches can lead to new insights into human sensorimotor performance. Moreover, a greater understanding of the factors underlying the wide range of performance capabilities seen across individuals can promote personalized medicine and refinement of rehabilitation strategies, which stand to be more effective than “one size fits all” treatments.

## Background

This paper provides a high level overview of the 2016 Biomechanics and Neural Control of Movement conference session on sensorimotor adaptation and learning. In the past few decades there have been substantial changes in perspectives of motor learning; predictive and optimal control theories have been put forth to explain how performance can be updated despite physiological limitations such as feedback delays and impedance. This forward modeling approach has been described by Miall and Wolpert [1]; the current state of the body is used as a starting point, and motor efference copy is used to predict action outcomes. One can therefore implement some required corrections without waiting for action feedback. Furthermore, error correcting mechanisms seem to leverage the same forward modeling processes that are used to plan and initiate voluntary actions, with hallmarks of corrections being visible at latencies as short as 60 ms [2, 3]. It has also been demonstrated that both forward models and error correction processes are modified with sensorimotor adaptation (cf. [4–7]).

In the past ten years, substantial progress has been made in identifying neurocognitive correlates of adaptation to sensorimotor perturbations and individual differences contributing to varying degrees of success with adaptation and learning. This paper provides a perspective on these topics. Specifically, section II outlines how the study of individual differences in learning and adaptation rates can yield understanding of the neural and cognitive processes underlying these behaviors. Section III highlights genetic approaches as another pathway to elucidating individual differences in learning. We then discuss clinical implications of the reviewed work (section IV) and future directions that may prove fruitful for further study (section V).

## Neurocognitive contributions to skill learning and sensorimotor adaptation

It has long been understood that semantic, episodic, and procedural memories differ in their characteristics and neural substrates; for a historical perspective see [8]. Many have interpreted this to mean that the acquisition of procedural memories—or knowing how to perform a skill—is implicit, occurring outside of awareness and not engaging overt cognitive resources. Implicit memories cannot be verbalized but rather are inferred to exist based on performance assessments. In contrast to this notion are several models and experimental findings supporting the conjecture that early motor learning and adaptation rely upon cognitive resources such as attention, working memory, and inhibition of competing motor plans. Fitts and Posner’s stages of learning model [9] purported that early skill learning relies on verbal self-talk. Indeed, Fitts has said “…sharp distinctions between verbal and motor processes, or between cognitive and motor processes serve no useful purpose” [10]. While classic work from Nissen and Bullemer [11] demonstrates that action sequences can be learned by Korsakoff’s patients, who have declarative memory impairments, the authors also found that attention is required for sequence learning. Thus, even when learning is implicit cognitive processes can play a role.

Sensorimotor adaptation has been studied by having individuals make movements while receiving distorted visual feedback of their actions [12–15] or while moving against a perturbing force field [16]. Adaptation involves adjusting motor commands on a trial by trial basis resulting in updated forward modeling processes and modifications of within trial corrective processes. Adaptation has traditionally been viewed as an implicit, obligatory process (cf. [17]). However, recent experiments support the view that both declarative (knowing what to do) and procedural (knowing how to do) processes contribute to sensorimotor adaptation [18–20].

Sensorimotor adaptation is thought to rely on at least two time varying processes [14, 21–24] which appear to engage differential neural systems and cognitive processes. For example, we and others have reported involvement of the right dorsolateral prefrontal cortex (DLPFC), dorsal anterior cingulate cortex, and parietal regions in early stages of adaptation [14, 23, 25, 26], with a shift towards cerebellar and parietal regions later in adaptation [15, 24, 27–29]. We have shown that individual differences in spatial working memory capacity and activation levels of the right DLPFC predict variations in the rate of adaptation across the first ~75 trials [14]. Moreover, short term fatigue of spatial working memory slows the rate of adaptation [30] and facilitation of right DLPFC with noninvasive brain stimulation speeds adaptation [31]. Recent work has demonstrated that explicit aiming strategies play a stronger role early in adaptation than was previously believed to be the case [20], and that spatial working memory capacity predicts individual differences in this explicit component of adaptation [32]. We and others have also shown that aging is associated with declines in the early phase of adaptation [23], a failure to engage spatial working memory and activate the right DLPFC [23], along with deficits in explicit memory [33]. Stroke patients with right frontal damage are impaired at making online corrections, suggesting that right DLPFC may a play a role in adaptive updating of corrective processes [26]. Studies documenting a correlation between adaptation of corrective actions and success with trial by trial modification of movement plans suggests that individual differences in forward models may contribute to both processes [5, 6].

These preceding examples support a role for cognitive processes in general, and spatial working memory specifically, early in sensorimotor adaptation. Moreover, they demonstrate the utility of investigating individual differences as a source of information rather than simply a reflection of noise. That is, identification of cognitive, neural, neurocomputational and/or genetic predictors of individual differences in adaptation rates can elucidate the mechanisms underlying adaptive sensorimotor behaviors.

Implicit and procedural processes govern behavioral change as adaptation progresses [20]. This slower phase of adaptation is thought to rely on motor corticostriatal and corticocerebellar networks [34]. For example, long term sensorimotor adaptation is associated with gray matter volumetric changes in the primary motor cortex (M1): Landi et al. [35] reported increased gray matter volume in the hand region of M1 after participants adapted manual aiming movements to distorted visual feedback in multiple practice sessions over one week. We have also recently reported increased gray matter volume in the motor and somatosensory cortical leg regions of astronauts after adaptation to the microgravity environment of space [36]. Christou et al. [32] have further reported that spatial working memory capacity does not predict faster adaptation when implicit processes dominate, suggesting reduced reliance on declarative processing and prefrontal cortex. It has also been demonstrated that stronger resting state cerebellar-thalamic connectivity is associated with faster adaptation later in practice [37].

Interestingly, once a learner reaches the state where representations are firmly procedural, performance can be disrupted by engagement of declarative processes. For example, Flegal and Anderson [38] have shown that verbalizing approaches to golf putting enhance performance for novices but impair that of more skilled golfers. In addition, knowing that one’s performance is being watched and evaluated by others has been shown to disrupt a previously well-learned action sequence [39]. Thus while explicit cognitive strategies may be important to kick-start adaptation, they can actually interfere with retention and implementation of well learned actions.

## Genetic analysis of individual differences in neuromotor adaptation and learning

Jack Adams is remembered for the breadth and depth of his contributions to the study of motor control and learning. In highlighting the challenges posed by idiosyncratic variations in behavior when one is seeking to resolve through experimental methods the enduring problems of human learning, he referred in characteristically colorful manner to the “individual differences that reside in the cesspool of the error term” (as cited in Schmidt, [40] p. 83). Elsewhere he expressed the view that explanatory models must first be concerned with general processes, rather than individual differences in these processes. The corollary was that the processes will be established by experimental research, and that only subsequently will variations among individuals be entered into the formulation [41]. The argument to be advanced here is essentially the reverse. It is that the study of individual differences exhibits the potential to contribute in a pre-eminent manner to resolving the processes that mediate human motor control in general, and human motor learning in particular. We have alluded to this approach in the preceding section of the paper, reporting associations between individual differences in brain activation levels or spatial working memory capacity and rate of learning. In this section, the case will be made that contemporary advances in genetic analysis provide the means to make good on this potential.

Perhaps curiously, our field of scientific enquiry has been largely impervious to many of the recent developments that have taken place in genetics and molecular biology. The past twenty years or so have seen the first human gene map established [42], the results of the Human Genome Project released ([43, 44], the endeavour being declared complete in 2003), the Map of Human Genetic Variation (HapMap) published [45], and the first results of the ENCODE project – which aims to identify all functional elements in the human genome, announced [46]. And yet the knowledge thus provided has had little obvious impact on the development of theories that concern the processes of motor control and learning. An effect around the periphery of our field can however be discerned. This has been made possible by one of the most obvious practical consequences of these large-scale scientific projects – the plummeting costs of genotyping. An assay for a single nucleotide polymorphism (SNP) can now be purchased for less than one hundred dollars, and thousands of individual samples can be processed in a matter of hours, each at the cost of a few cents. Along with this capability however, comes the responsibility of exploiting the obvious scientific potential in a focused manner – ideally motivated by clear a priori hypotheses. This requirement is particularly important in the domain of motor control and learning, as our characteristic sample sizes are a tiny fraction of those required by genome wide association studies (GWAS). Indeed, even in areas of research for which very large cohorts are the norm, the GWAS approach has been plagued by extremely low levels of replication (e.g. [47]) which, in spite of the fact that the reasons are broadly recognised and discussed (e.g. [48–50]), remains a pervasive problem (e.g. [51, 52]).

It is however possible to undertake hypothesis driven research whereby the range of potential genetic variations under consideration is constrained in advance by, for example, extant disease and animal models, or by knowledge that has been derived in cognate domains – i.e. the impact of particular genotypic variations on related phenotypic expression (e.g. upon other forms of learning). The motivating assumption is typically that, if individual variations in the transcription of a gene or of a system of genes account for a substantial portion of observed differences in the expression of a phenotype (e.g. rate of motor learning), it can be inferred that physiological processes regulated by the product of that gene (or system of genes) play a determining role.

This is perhaps best illustrated by a concrete example. Pearson-Fuhrhop et al. [53] examined the influence on motor learning of five genetic polymorphisms with established effects on dopamine neurotransmission, using a sequencing task that placed a particular onus upon manual dexterity. The putative influence of individual variations in three dopamine receptor genes (DRD1, DRD2, and DRD3), and two genes for dopamine degradation enzymes (catechol- O-methyltransferase - COMT and DAT), were combined in a gene score. The contribution of specific polymorphisms (i.e. of the five genes) to the combined gene score was determined by prior knowledge of their effect on dopamine neurotransmission (as assessed in the context of cognition and learning). Individuals with the highest gene score – interpreted as greater endogenous dopaminergic neurotransmission, exhibited superior performance of the task following a two weeks training period. While it may not appear initially that findings of this nature contribute much that is additional to our understanding of the processes that mediate motor learning (beyond confirmation that dopamine neurotransmission plays a role), decomposition of the gene score indicated that individual variations in the DRD2 receptor gene were particularly influential in the context of this particular form of learning. Alternative outcomes could however have been conceived. In motor learning tasks that differ along several dimensions from the one employed by Pearson-Fuhrhop et al. [53], instrumental roles have been ascribed to other aspects of dopamine function.

For example, Noohi et al. [54] examined the potential impact of SNPs of the COMT and DRD2 genes upon the characteristics of initial motor learning (i.e. in a single training session), as assessed using a visuomotor adaptation task, and a sequence learning task. Although individual variations in COMT genotype were associated with differences in rates of visuomotor adaptation, this was not the case for genotypic variations in DRD2. The extent to which the motor sequences were learned, did not however appear to be influenced by individual variation in either the COMT or the DRD2 gene. The point is therefore that the relative functional contributions to motor learning of processes regulated by specific gene products are not equivalent across motor tasks. By the same token, elucidating the degree to which the characteristics of motor learning (or indeed motor control) are subject to the influence of individual differences in the expression of particular genes or systems of genes, informs our understanding of the specific cellular processes that are involved. In so much as different neural systems vary with respect to cellular mechanism (e.g. principal neurotransmitters), it may also be the case that lifespan variations in the degree to which salient genotypic variations influence behavioral outcomes have the potential to reveal age-related changes in functional brain architecture (e.g. [55]).

Of all the genetic variants that have been investigated with a view to determining their influence on motor control and learning, brain derived neurotrophic factor (BDNF) is perhaps most prominent. BDNF is one of the classic neurotrophins discovered first in the 1950s. It is expressed as proBDNF, a precursor peptide that is cleaved to generate the mature protein [56]. There is sufficient evidence to conclude that BDNF affects neurogenesis, synaptogenesis, synaptic transmission and certain aspects of cognitive function. Although several SNPs in the gene encoding BDNF have been identified, in the vast majority of studies that have focused on genotypic variations in its expression, attention has been directed to a substitution of valine to methionine at position 66 (Val66Met) in the prodomain. In a seminal investigation, Kleim et al. [57] examined the impact of repetitive movements of the index finger on the area of the scalp from which motor potentials (MEPs) could be evoked in the first dorsal interosseus (FDI) muscle by transcranial magnetic stimulation (TMS). It was reported that individuals homozygous for the Val allele exhibited increases in the area of the scalp from which MEPs could be elicited following the repetitions of movement, that were greater than those present in individuals possessing either one or two Met alleles. The amplitude of the MEPs obtained from the former group also increased to a greater degree than was the case for the MET carriers. Although there has since followed a proliferation of reports concerning the potential influence of this specific BNDF polymorphism on short-term changes in the performance of motor tasks, there have been remarkably few instances in which retention (or transfer) tests have been used to assess whether there is a commensurate effect on motor learning (e.g. [58, 59]). And in this regard the outcomes are equivocal. Relatedly, and contrary to what customarily appears to be assumed, there is also very little evidence to support the assertion that the BDNF val66met polymorphism influences responsiveness to therapy following stroke [60].

Thus, while on the basis of the well-characterized influence of BDNF on neurogenesis, synaptogenesis, and synaptic transmission (derived largely from animal models), the expectation that individual variations in its expression should provide useful information concerning the processes that mediate motor learning seems entirely reasonable, there is presently little supporting empirical evidence. A key problem in this regard is that almost every study conducted thus far has been dramatically underpowered [61]. In order to achieve adequate statistical power in testing a single SNP, 248 cases are typically required [62]. On the basis of most extant research, it is therefore impossible to ascertain the true size of any effect that may be present. Similarly, it is a challenge to determine whether the positive associations between BDNF genotypes and some aspects of motor function that have been reported on occasion reflect false positives (i.e. type I errors) or faithfully represent the presence of a real effect. It is worth noting that in a number of cognate domains in which larger sample sizes and multiple replications have been the norm, cumulative meta-analyses have been consistent in revealing shrinkage in the size of the effect attributable to BDNF genotypic variations in the period following the first (positive) reports (e.g. [63–65]). This is not a characteristic that is restricted to the study of BDNF. In many domains in which candidate genes have been identified on an a priori basis (i.e. rather than using a GWAS approach) there are extremely poor rates of replication (e.g. [66]).

Can the reasons for this state of affairs be identified and ameliorated, or is the potential of genetic analysis offered at the start of this section merely a chimera? There are certainly problems arising from the use of animal models to identify candidate genes. It is becoming increasingly apparent that there are pronounced differences in the RNA expression profiles of specific genes even across various regions of the human brain ([67]). Such findings put in perspective the (perhaps often implicit) assumption that the cellular action of a gene or system of genes in relation to neural function is conserved across species along with its presence. It is similarly clear that we need to move away from an almost exclusive focus on variations in the protein coding regions of the genome to encompass consideration of regulatory elements that control gene expression (e.g. [67]). The most critical general requirement in our field of enquiry is however a dramatic increase in sample sizes [68] – a point that has been made emphatically and eloquently elsewhere [61]. Indeed power analyses lead to the inescapable conclusion that if there are two ways to measure phenotypic expression (e.g. of motor learning) – a high-reliability variant for which only limited sample sizes can be obtained due to the demands on time, effort and other resources, versus a low-reliability variant for which large sample sizes become feasible, the latter represents the best strategy in genetic analysis [48]. While such an approach may seem an anathema to those of us trained in the laboratory traditions of motor control, biomechanics or exercise physiology, there remain reasons to believe that an effectual balance may be struck. On the one hand there will be many small individual gene effect sizes for traits not under strong directional selection, and extremely large data sets will be required for their detection [48]. On the other hand, there is sufficient evidence to indicate that the effect sizes associated with certain genes and gene systems (relating to dopamine neurotransmission, for example) are such that some of the cellular processes implicated in human motor control and learning may be resolved reliably using sample sizes in the order of hundreds of participants [69, 70]. The foregoing caveats notwithstanding, it seems reasonable to conclude that although genetic analysis is likely to remain on the periphery of our field in the immediate future, it exhibits the potential to make important contributions to our understanding of processes that mediate human motor control in general, and human motor learning in particular.

## Leveraging sensorimotor adaptive processes and individual differences for clinical benefits

Laboratory tasks to study motor control and sensorimotor adaptation can seem rather contrived, but they do have relevance to multiple conditions. For example, astronauts must adapt their movement control to the altered vestibular inputs that occur in the absence of Earth’s gravity, and they exhibit aftereffects for this adaptation upon return from space (cf. [71]). A greater understanding of the underlying mechanisms of adaptive processes and behavioral or genetic markers of individual differences in success of adaptation can lead to predictors of adaptability [71]. Identifying which individuals exhibit propensity for slower learning and adaptation can lead to individually targeted training and rehabilitation approaches [72].

Hemispatial neglect is a condition that can follow unilateral brain damage, resulting in attention and awareness deficits on one side of space/the body. Interestingly, sensorimotor adaptation to laterally displacing prism lenses has been shown to be effective at ameliorating the symptoms of neglect [73, 74]. Moreover, the aftereffects of adaptation to walking on a split-belt treadmill in which the two belts move at different speeds can improve symmetry of walking in stroke patients [75]. Here as well a better understanding of the underlying processes of sensorimotor adaptation and predictors of individual differences in success can lead to optimized treatment approaches.

## Future directions

It is worth noting that much of the literature discussed in this article addresses visuomotor adaptation; only a few examples of force field adaptation or skill learning are included. It is difficult to incorporate the devices used for force field adaptation into the MRI environment, although it has been done successfully for both fMRI [76, 77] and PET [78, 79]. Regardless, to have a more complete view of the biological bases of motor learning broadly defined, additional studies are required.

We also advocate further investigation of individual differences to better understand motor control and learning; such variation reflects not only measurement noise but also meaningful information regarding predictors of successful learning and performance. This approach requires interdisciplinary teams to bring modern techniques to bear on questions of motor control. Further, identifying predictors of individual differences requires large sample sizes with diverse performance levels and replication in independent samples. Interestingly, research in the cognitive domain has repeatedly demonstrated that associations between targeted SNPs and behavior increase with advancing age, when neural processes are in decline [80, 81]. If the same holds for motor control, genetic markers may provide a route to predict motor declines and loss of independence in older adults.

## Abbreviations

BDNF: brain derived neurotrophic factor
COMT: Catechol-O-methyltransferase
DLPFC: Dorsolateral prefrontal cortex
DRD1: Dopamine receptor D1
DRD2: Dopamine receptor D2
FDI: First dorsal interosseous
GWAS: Qenome wide association screen
M1: Primary motor cortex
MEP: Motor evoked potential
SNP: Single nucleotide polymorphism
TMS: Transcranial magnetic stimulation
