# Correction to: Sensorimotor learning: Neurocognitive mechanisms and individual differences

**DOI:** 10.1186/s12984-017-0311-5

**Published:** 2017-10-02

**Authors:** R. D. Seidler, R. G. Carson

**Affiliations:** 1 University of Florida, P.O. Box 118205, Gainesville, FL 32611-8205 USA; 2 Trinity College Dublin, Dublin, Ireland; 3 Queen’s University Belfast, Belfast, Ireland

## Erratum

The original article [1] mistakenly omits grant support information.

As such, the authors would like to acknowledge the following funding source:

NSF award CBET 1644835.
